# Generalized Poincaré plots analysis of heart period dynamics in different physiological conditions: Trained vs. untrained men

**DOI:** 10.1371/journal.pone.0219281

**Published:** 2019-07-05

**Authors:** Mirjana M. Platiša, Tijana Bojić, Sanja Mazić, Aleksandar Kalauzi

**Affiliations:** 1 Institute of Biophysics, Faculty of Medicine, University of Belgrade, Belgrade, Serbia; 2 Laboratory of Radiobiology and Molecular Genetics, Institute of Nuclear Sciences "Vinča", University of Belgrade, Belgrade, Serbia; 3 Institute of Medical Physiology, Faculty of Medicine, University of Belgrade, Belgrade, Serbia; 4 Department for Life Sciences, Institute for Multidisciplinary Research, University of Belgrade, Belgrade, Serbia; Universidade Federal de Juiz de Fora, BRAZIL

## Abstract

**Background:**

Recently we proposed a new method called generalized Poincaré plot (gPp) analysis which gave a new insight into the pattern of neurocaridac control. In this study we examined potential of gPp method to reveal changes in cardiac neural control in young athletes during three conditions: supine rest, running and relaxation, with respect to untrained subjects.

**Methods:**

This method is based on the quantification of Pearson’s correlation coefficients *r*(*j*, *k*), between symmetrical (*j* = *k*) and asymmetrical summed *j* previous and *k* following RR intervals up to the 100th order (*j*,*k*≤100).

**Results:**

Differences between groups were obtained at all levels of this analysis. The main result is the significant difference of *NAI*, normalized index of asymmetry, between groups in running, which was originated in different positions of local maxima of *r*(*j*, *k*). Compared with untrained subjects, these findings indicate modified neural control and altered intrinsic heart rate behavior in athletes which are related to some kind of memory mechanism between RR intervals.

**Conclusion:**

Obtained results provide great potential of gPp method analysis in the recognition of changes in neurocardiac control in healthy subjects. Further studies are needed for identification of altered cardiac regulatory mechanisms whose background may be useful in the evaluation of genesis of athletes neurocardiovascular pathology.

## Introduction

Methods of time series analysis developed in last few decades enable recognition and quantification of the behavior of regulatory mechanisms in complex dynamics of physiological systems. In heart rate variability (HRV) analysis linear and nonlinear methods are applied with various assumptions from periodical behavior to stochastic nature of time series. In few past decades, their applications lead to significant knowledge about changes of regulatory mechanisms under various physiological and pathological conditions [1]. More, new methods analyze the structure of interbeat intervals time series and their interrelations on scales with different length to reveal network of different regulatory mechanisms which operate over different time scales [2,3].

A recently developed generalized Poincaré plot (gPp) analysis of heart period data gives a new insight into the dynamics of cardiac neural mechanisms [4]. It is based on the computation of summed durations of *j* previous and *k* following consecutive RR intervals and quantification of their relationship by Pearson correlation coefficients of matrices for *j*, *k* = 1, …, 100 intervals. When symmetric (*j* = *k*) gPp was calculated for previous *k* and following *k* intervals, integral dynamics of heart beat intervals appeared as specific trajectories in healthy subjects, instead of well known scattergrams resulting from classical Poincaré analysis. Expanded gPp, i.e. calculation of relationship between *j* and *k* summed RR intervals when *j* ≠ *k*, gave us an insight into the relationship between previous *j* and following *k* intervals, revealing new properties of heart interval dynamics. It was quantified by the index of asymmetry and visually supported by three-dimensional space distribution of local maxima of Pearson correlation coefficients, *r*(*j*, *k*).

Exercise treadmill stressing test is a cardiovascular testing which is mostly used to determine cardiac functional capacity [5, 6]. During the final phases of exercise, to the exhaustion, sympathetic discharge is maximal and parasympathetic stimulation is withdrawn, resulting in autoregulation with generalized vasoconstriction. In relaxation, during post exercise phase, vagal drive returns to baseline, and it is more pronounced in well-trained athletes. Morphological and functional remodeling of autonomic nervous system is a well known phenomenon in athletes [7, 8]. Athletes are also the group carrying a special risk for the appearance of neurocardiovascular syndromes like atrial fibrillation (AF) [9, 10] and sudden cardiac death [11, 12, 13]. Both pathophysiological phenomena are the consequence of dual change—morphofunctional change of the heart and proarrhythmic diathesis of autonomic nervous system [13]. GPp method was presented as the method powerful enough to reveal differences in intrinsic neural mechanisms of AF patients with respect to healthy group [4]. In accordance with that finding we applied this method in order to test the ability of gPp to characterize the profile and trace the arrhythmogenic diathesis of heart rate autonomic nervous control in athletes.

The design of gPp analysis is applied to examine its potential to quantify differences in neural cardiac control in three different physiological conditions as well as differences in cardiac neural control between trained and untrained subjects.

## Methods

### Experimental protocol and data acquisition

The experimental study was conducted in accordance with the Declaration of Helsinki. Written informed consent was obtained from all participants. The protocol of this study was approved by the Ethics Committee of the Faculty of Medicine, Belgrade University (Ref. Numb. 29/XII-18). All subjects were healthy with no history of any respiratory or cardiovascular diseases. Consumption of coffee, tea, alcohol or a heavy meal within a few hours prior to the test was not allowed and no physical exercise was permitted the day before. All subjects were tested in an environmentally controlled Exercise Physiology Laboratory (21–23°C) between 9:00–11:00 a.m. during 2 months (April and May 2007) at the Institute of Medical Physiology, Faculty of Medicine, Belgrade University [14]. Nine well-trained basketball players (mean age 18; range 17–22) were at least five years active players (with 15 hours of training per week). Control group of 11 untrained male subjects (mean age 23; range 19–26) was formed from physically inactive medical students (with less than 5 hours of training per week). There was no statistical difference between body mass index (23.1 ± 0.6 kg/m^2^ and 24.5 ± 0.6 kg/m^2^) and fat-free mass (78 ± 3 kg and 73 ± 3) kg of trained and untrained subjects, respectively. ECG data were collected during the following study design with quantification of 2440 nV/bit and the sample rate of 240 Hz (using Viasys with integrated 12-lead ECG, Jaeger, Germany). Initially subjects were at rest in a supine position for 30 minutes. Then, they were asked to stand on a fixed treadmill and after 5 min they performed a progressive running exercise test on treadmill (Viasys, Jaeger, LE 200 CE, Germany). The incremental running test began with an initial speed of 9 km/h and initial incline of 2%. The incline successively increased by 2% in periods of 3 minutes until the subjects stopped due to exhaustion. The test was finished when subject achieved the maximal heart rate or due to volitional exhaustion [14]. Immediately after the running, subjects were relaxed in supine position for 15 minutes. The RR intervals of normal sinus rhythm were extracted from ECG recordings using tools in Origin 6.0 (Microcal, USA). For further analysis we used epochs of 1024 RR intervals for three physiological states: supine rest, running and relaxation (S1 Appendix). For relaxation (recovery) we selected period from the 5^th^ minute after finished running phase. We calculated mean RR interval and SDRR (standard deviation of RR intervals) for every subject in all three physiological conditions.

### Generalized poincaré plots

All analyses were performed using our original programs developed within MATLAB (MathWorks Inc., Natick, MA01760-2098, United States) [4]. With *RRn*−*j* we denote summed duration of previous successive *j RR* intervals, while *RRn*+*k* refers to the analogous duration of the next successive *k* intervals. They were calculated by adding the durations of the corresponding intervals around a particular observed *R* wave sliding along the ECG signal. Naturally, if the ECG signal contained a total of *N* points of *R* waves, only *N–j–k* points could be drawn in this way. If the analysis was performed with a pair of intervals (*j*,*k*), the term “gPp of the order (*j*,*k*)” was proposed. For each gPp order, a separate scattergram could be drawn and the corresponding Pearson's coefficient of linear correlation, *r*(*RRn-j*,*RRn+k*), briefly denoted as *r*(*j*,*k*), could be calculated. In our previous work [4] we described the emerging properties of these scattergrams which appeared in healthy subjects and which were different from either classical Poincaré plots, or from scattergrams of AF patients. Further we observed *r*(*j*,*k*), obtained for all included values of *j* and *k*, as matrices and concentrated on the asymmetry of their element values, since this property appeared to be sensitive to the subjects cardiac health condition. In order to quantify this asymmetry, we introduced a normalized asymmetry index (*NAI*) for a *m* × *n* type matrix

$$NAI=\frac{1}{m\times n}\frac{1}{\bar{r}}\sum_{j=1}^{m}\sum_{k=j+1}^{n}\left(r(k,j)-r(j,k)\right)$$ (1)

where *r*(*j*,*k*) is the matrix element, while

$$\bar{r}=\frac{1}{m\times n}\sum_{j=1}^{m}\sum_{k=1}^{n}\left|r(j,k)\right|.$$ (2)

This specific type of normalization allowed us to compare asymmetry indexes from matrices of different range of their element values and different sizes. We also studied potential existence and positions of local maxima of *r*(*j*,*k*), since each local correlation maximum could be linked to a time interval in which a neurocardiac regulatory mechanism is operating. All *r*(*j*,*k*) matrices and their *NAI* values were calculated from epochs with 1024 RR intervals.

### Statistical analysis

The normality of the distribution of the data was tested by Shapiro-Wilk test. Repeated measures one way analysis of variance was applied to assess the significance of the differences between the three physiological conditions in the group (pairwise comparison). Including the group effect we examined existence of a significant interaction between the group and condition effect. For comparison between data for trained and untrained subjects in particular conditions, the independent samples *t* test was used. The data are given as mean values ± standard errors. A value of *p* < 0.05 was considered statistically significant.

## Results

**Fig 1:**
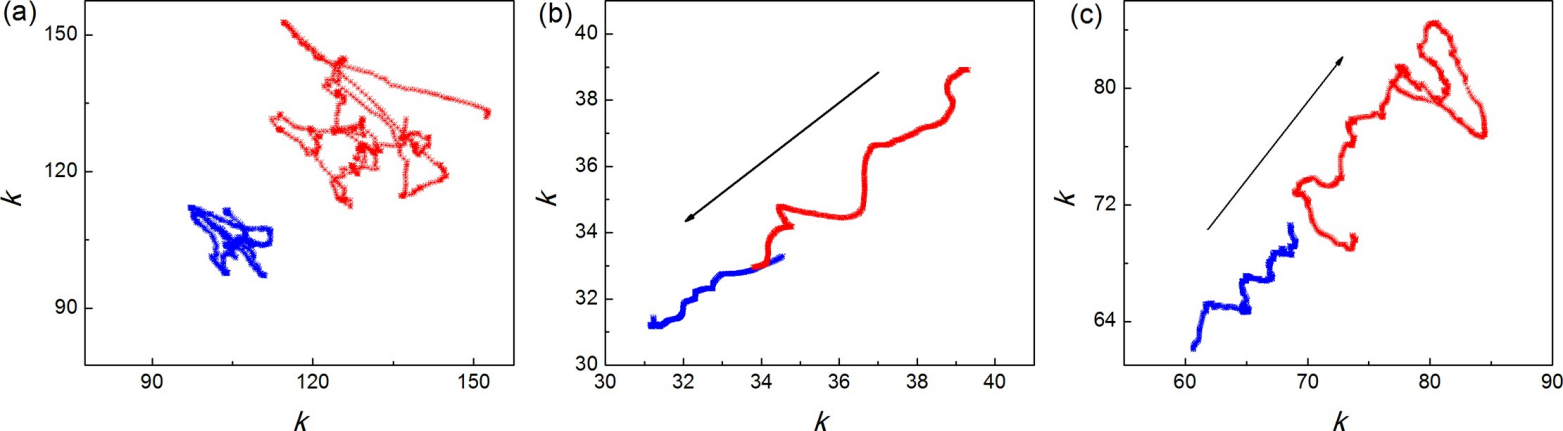
Trajectories of summed duration of 100 RR intervals. Before (abscissa) and after (ordinate) an observed sliding RR interval (in seconds) in trained (red) and untrained (blue) subjects at (a) supine rest, (b) running and (c) relaxation. Arrows indicate the direction of movement of shorter RR intervals; (b) entering the analysis and (c) exiting the analysis. Note different scales of both axes in different states.

Examples of trajectories of summarized RR intervals obtained by gPp method of the symmetrical 100^th^ order for one trained and one untrained subject at supine rest, running and relaxation are presented on Fig 1.

**Fig 2:**
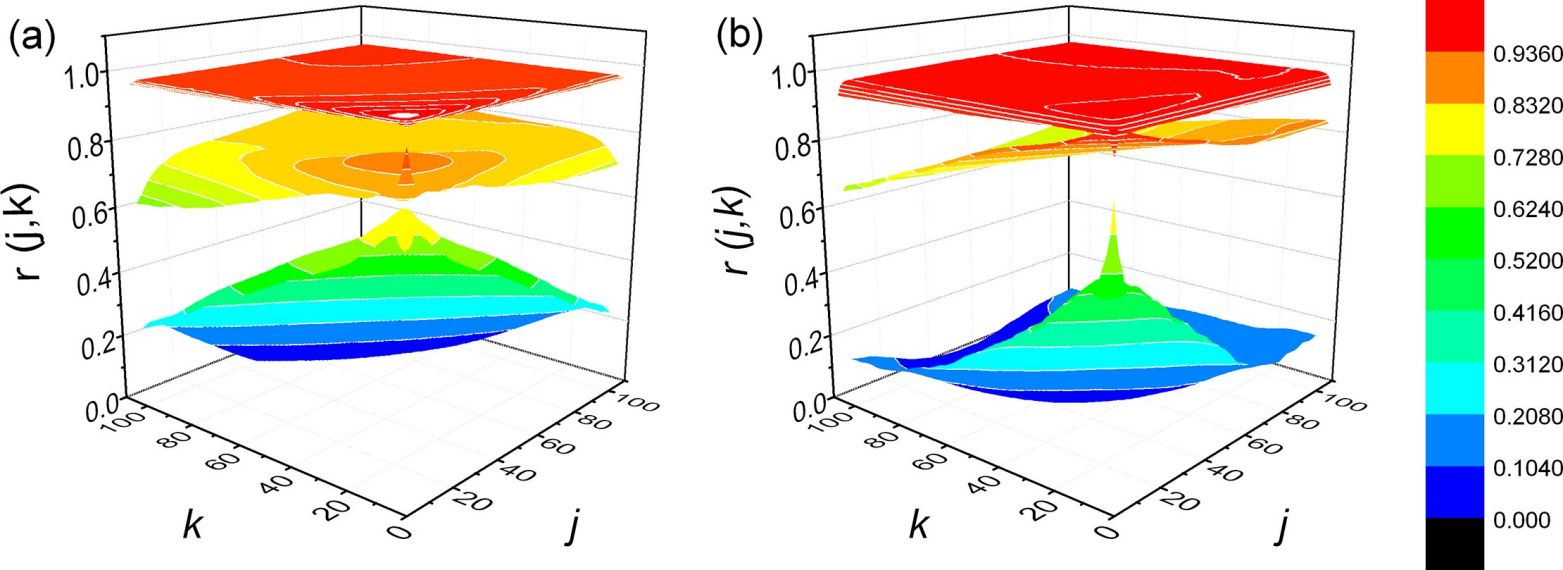
Examples of matrices of Pearson’s coefficients *r*(*j*, *k*). (a) in one trained subject and (b) one untrained subject. In both examples, lower matrices were obtained at supine rest, middle at relaxation and upper at running.

For trained and untrained subjects state of supine rest is characterized by specific hanks, while in running they almost disappear. During relaxation, 5 minutes after they stopped running, trajectories in the shape of elongated hanks were obtained in both subjects. In trained subject, hank is more complex than in untrained man. After the symmetrical analysis where *j* = *k*, we applied expanded gPp analysis for *j*≠ *k* and calculated matrices of Pearson’s coefficients *r*(*j*, *k*) (Fig 2, S2 Appendix) and their indexes of asymmetry (*NAI*). We found significant interaction between group effect and state effect on *NAI* (*p* = 0.048). Results of descriptive statistics of *NAI*, RR and SDRR with comparisons between conditions in every group are presented in Table 1.

**Table 1:** Comparison of SDRR and *NAI* between three physiological conditions in groups of untrained and trained subjects.

		Supine rest (1)	Running (2)	Relaxation (3)	*P* (1–2)	*P* (1–3)	*P* (2–3)
**UT**	***NAI***	-0.019 ± 0.012	-0.0038 ± 0.0013	-0.0048 ± 0.0084	0.28	0.33	0.92
	**RR (s)**	0.860 ± 0.045	0.3281 ± 0.0053	0.574 ± 0.013	0.01	0.01	0.01
	**SDRR (s)**	0.0731 ± 0.0090	0.0172 ± 0.0024	0.0298 ± 0.0050	0.01	0.01	0.07
**T**	***NAI***	0.0052 ± 0.0088	(5.6 ± 16) 10^−5^	-0.0180 ± 0.0063	0.58	0.04	0.02
	**RR (s)**	1.029 ± 0.048	0.3481 ± 0.0061	0.705 ± 0.021	0.01	0.01	0.01
	**SDRR (s)**	0.121 ± 0.020	0.0168 ± 0.0020	0.0418 ± 0.0040	0.01	0.01	0.01

Mean values ± standard errors. UT–untrained, T–trained. SDRR–standard deviation of RR intervals, *NAI*–normalized asymmetry index.

**Fig 3:**
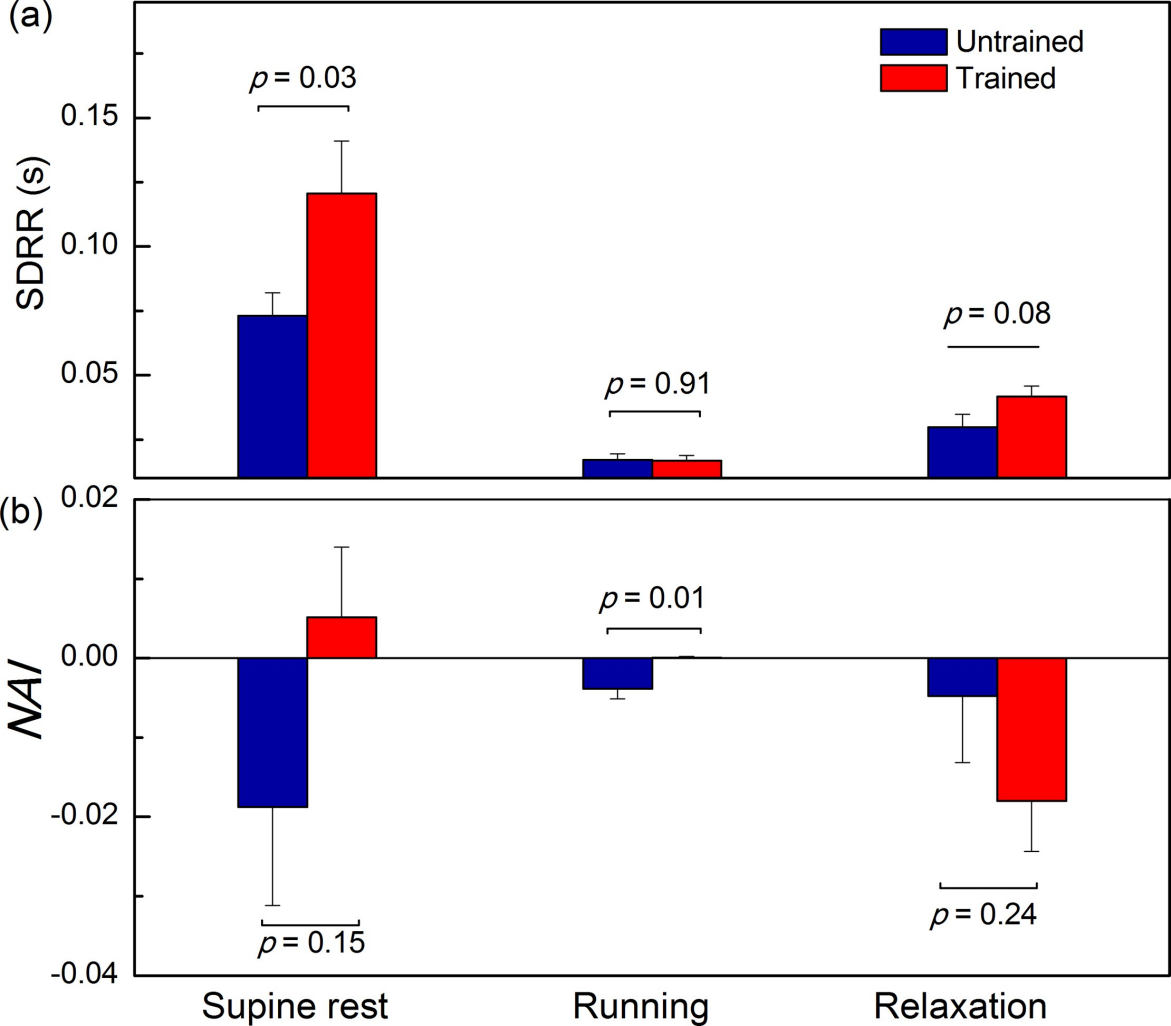
**Bar graphs show (a) SDRR—standard deviation of RR interval series and (b) *NAI***–**normalized asymmetry index**. Data are presented as mean with standard errors. Statistical comparison of mean values between untrained and trained subjects during supine rest, running and relaxation.

Statistical significances of differences between groups in all three conditions are presented on Fig 3A for SDRR and on Fig 3B for *NAI*. During running there was no statistical difference between SDRR (Fig 3A) but *NAI* was statistically lower in untrained than in trained subjects (Fig 3B).

**Fig 4:**
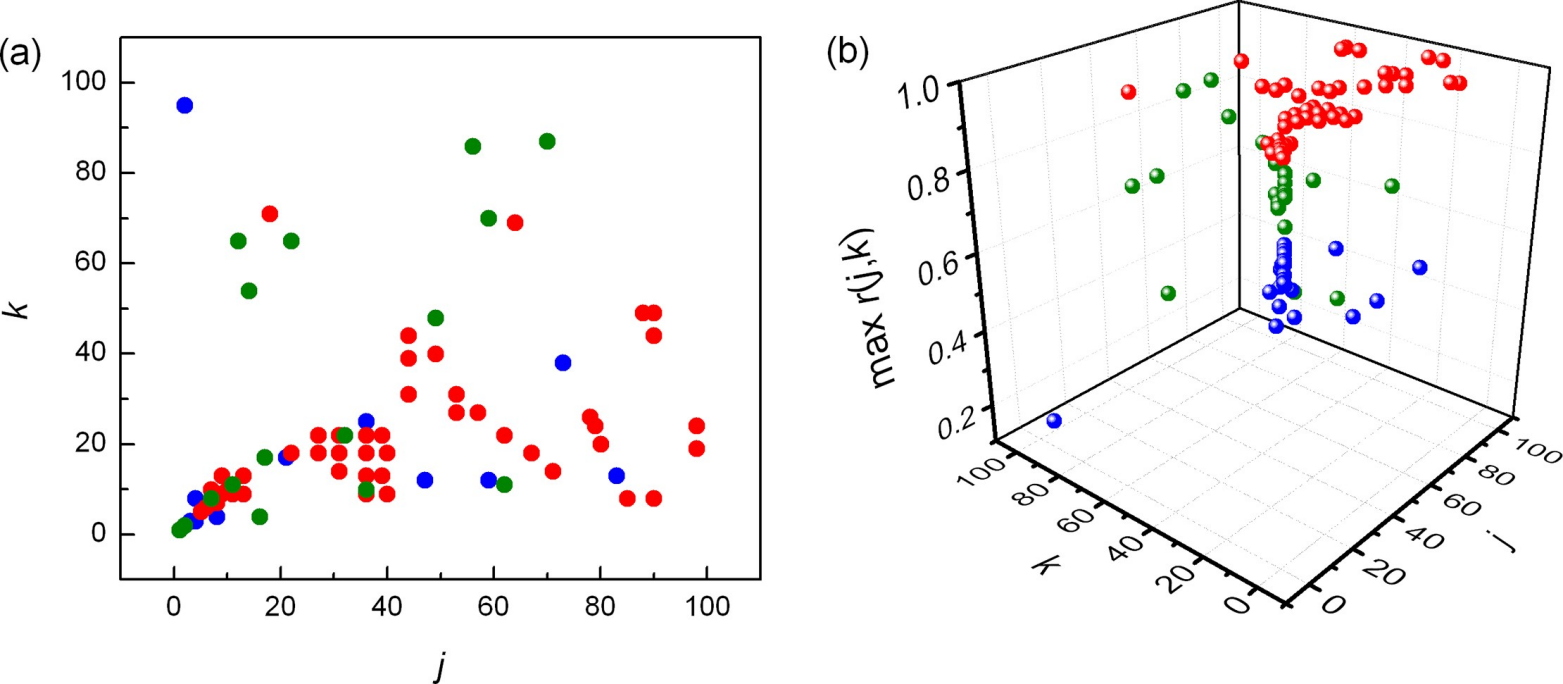
**Distribution of pooled local maxima of Pearson's correlation coefficients matrices (max *r*(*j*, *k*)) in trained subjects at supine rest (blue), running (red) and relaxation (green)**. (a) projected on the (*j*,*k*) plane, (b) plotted in 3D.

**Fig 5:**
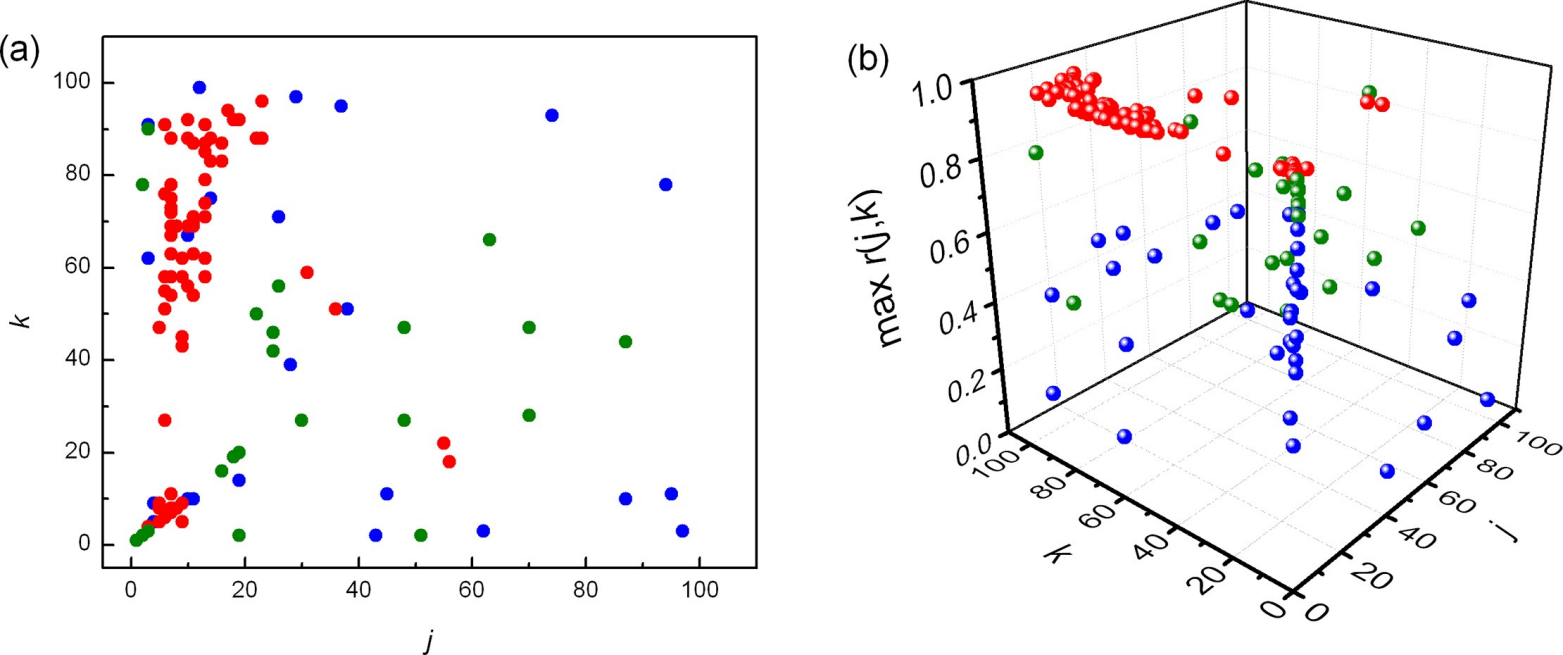
**Distribution of pooled local maxima of Pearson's correlation coefficients matrices (max *r*(*j*, *k*)) in untrained subjects at supine rest (blue), running (red) and relaxation (green)**. (a) projected on the (*j*,*k*) plane, (b) plotted in 3D.

In untrained subjects we did not find significant differences for *NAI* between physiological states, but in trained group the difference were significant, except between supine rest and running (Table 1). Mean value of *NAI* obtained for relaxation was significantly smaller than *NAI* at running or supine rest. Further, we plotted positions of pooled local maxima of Pearson's correlation coefficients matrices *r* (*j*, *k*) (Figs 4 and 5).

Different positions of local Pearson's maxima during running in trained and untrained subjects are evident. In trained subjects dominant maxima were found for larger *j* and smaller *k*, while in untrained subjects it was opposite, dominant maxima were located over smaller *j* and larger *k*. This result indicated different behaviors of cardiac intrinsic and regulatory mechanisms during running in investigated groups.

## Discussion

Generalized Poincaré plot was recently proposed as a method for evaluation of dynamic in autonomic neural control mechanisms of heart rate [4]. In our view this method has a potential for the recognition of patterns of heart rate neural control mechanisms and their alterations in individuals with established patho/physiological conditions.

In our previous work we proposed that trajectories of summed duration of RR intervals (organized as “hanks”) represent different regimes of integral heart rate control, and they are specifically visible in basal metabolic condition (supine resting state). Our results are in accordance with data in the literature that autonomic neural control systems in different physiological conditions operate under different regimes [15, 16, 17, 18, 19]. This is valid also for athletes with respect to untrained subjects [7, 8, 20]. In basal conditions, the regulation of heart rate in athletes has a lower set point, due to parasympathetic predominance, while the heart rate set point in untrained subject is on the higher level [20, 21]. Our data (Fig 1) are in complete accordance with these facts: in basal conditions we can observe two hanks—the one organized around lower values of HR, having consequently higher values of summed RR intervals, belonging to the athletes, while the one organized around higher HR values, with higher basal sympathetic tone, belongs to untrained subjects. Specific situation happens in the running conditions: the trajectories of summed duration of RR intervals are elongated where athlete’s trajectory is above trajectory for untrained subjects.

In the running conditions, oscillatory nature of sympathetic nervous system that dominates heart rate control in running is abolished and in this condition of steady-state its nature becomes more linear with a high absolute value [22]. This is also in accordance with the elongated shape of the trajectories. The reappearance of hanks of summed duration RR intervals trajectories in relaxation speaks for the recovery of oscillatory nature of sympathetic nervous system and the recovering of parasympathetic activity. The fact that in relaxation both pathways (athletes and untrained subjects) are similar suggests that the mechanisms active during recovery have similar origin in both investigated groups [23, 24, 25]. However, trajectories in trained subjects have more complex hanks and probably they appear as the result of faster recovery of parasympathetic activity.

The fact that the trajectories have similar relationship with respect to all three conditions is in accordance with the recent hypotheses. Namely, the resting bradycardia as well as the bradycardia provoked by exercise training and following relaxation are also a consequence of pacemaker cells alteration and intrinsic heart rate reduction [14, 26, 27, 28, 29]. Hence, trajectories in trained and untrained subjects are a result of intrinsic electrophysiological changes in the sinus node associated with changes in the vagal and sympathetic activity.

In our previous study we suggested that the physiological background of *NAI* indexes might be a memory mechanism, specific also for the autonomic nervous system [22]. It is obvious that this mechanism shows an important difference between athletes and untrained subjects. The memory pattern expressed as *NAI* in supine condition has a positive value for athletes and negative value for untrained subjects. This relation is the same in running conditions, while it dramatically changes in relaxation, where *NAI* of athletes also becomes negative, more profoundly than in untrained subjects. Having in mind the fact that arrhythmias and sudden cardiac death, the pathologies that strike the athletes more often than untrained subjects, happen more often in the phases of rest and recovery with respect to the phase of physical activity [13], we suggest that at least one of the reasons could be the different *NAI* profile, that is notably different between athletes and untrained subjects in critical conditions.

The clusters of pooled local maxima of Pearson’s correlation coefficient also support the finding that different neural regulatory patterns are present in athletes and untrained subjects. It is of note that the cluster of Pearson’s coefficients of untrained subjects in running condition is less dispersed with respect to the same cluster of the athletes, suggesting less adaptable and less trained full blown sympathetic action on heart rate, typical for the untrained subjects.

## Conclusions

Our analysis for the first time addresses some important questions on the nature and dynamic features of autonomic nervous system in athletes with respect to untrained subjects. The data are also in line with the basic findings of exercise physiology and offer new insight into potential hazardous neuroplastic changes that occur during the professional physical training. An important caveat of this study is that physiological interpretation of the specific roles of parasympathetic and sympathetic nervous systems is based only on the comparison with the literature data. Even though we obtained reasonable concordance with the data in the literature, we believe that further pharmacological evaluation is necessary for the definite and unequivocal interpretation of gPp analysis results.

## Supporting information

S1 Appendix Raw data. (ZIP)

S2 Appendix Pearson matrices 100x100. (ZIP)
